# Verification of Resveratrol Inhibits Intestinal Aging by Downregulating ATF4/Chop/Bcl-2/Bax Signaling Pathway: Based on Network Pharmacology and Animal Experiment

**DOI:** 10.3389/fphar.2020.01064

**Published:** 2020-07-10

**Authors:** Tian-hao Liu, Wan-qing Tu, Wen-cong Tao, Qiu-er Liang, Ya Xiao, Li-guo Chen

**Affiliations:** College of Chinese Medicine, Jinan University, Guangzhou, China

**Keywords:** resveratrol, aging, network pharmacology, animal experiment, apoptosis, mechanisms

## Abstract

Resveratrol is one of the most well-known drugs used in the treatment of aging. However, the potential mechanisms of resveratrol on intestinal aging have not yet been fully investigated. Herein, we aimed to further explore the pharmacological mechanisms of resveratrol as a therapy for intestinal aging. We performed network construction and enrichment analysis *via* network pharmacology. Then a further animal experimental validation containing 20 female C57BL/6J (wild type, WT) and 16 female *ATF4^+/-^* (knock down, KD) naturally aging mice and oral supplementary resveratrol (44 mg/kg/day) for 30 days were conducted. The expression of superoxide dismutase (SOD), glutathione peroxidase (GSH-Px), catalase (CAT), linear alkylethoxylate (AE), and malondialdehyde (MDA) were measured by ELISA, the observation of pathological changes and apoptosis in intestinal tissue were performed by HE, PAS, and TUNEL staining, the ATF4/Chop/Bcl-2/Bax signaling pathway-related proteins and mRNAs expression were measured by western blotting and real-time PCR. The network pharmacology showed 132 targets of resveratrol on aging. The enrichment analysis showed resveratrol antiaging involved mainly included protein heterodimerization activity, apoptosis, etc. Then ATF4/Chop/Bcl-2/Bax signaling pathway in biological process of apoptosis was selected to verify the potential mechanisms. Animal studies showed resveratrol upregulated the relative expression of SOD, GSH-Px, CAT, AE, whereas it downregulated the relative expression of MDA in intestine compared with the control group. There was also higher relative expression of SOD, GSH-Px, CAT, AE, and lower relative expression of MDA in KD mice than that in WT mice. Moreover, there was higher relative expression of SOD, GSH-Px, CAT, AE, and lower relative expression of MDA in KD mice than that in WT mice after resveratrol treatment. Decreased ATF4, Chop, Bax but increased Bcl-2 proteins and mRNAs expression were determined after resveratrol treatment compared with the control group; lower ATF4, Chop, Bax but higher Bcl-2 proteins and mRNAs expression were found in KD mice than that in WT mice. Additionally, lower relative proteins and mRNAs expression of ATF4, Chop, Bax and higher relative expression of Bcl-2 in KD mice than that in WT mice after resveratrol treatment. These findings demonstrated that resveratrol substantially inhibited intestinal aging *via* downregulating ATF4/Chop/Bcl-2/Bax signaling pathway.

## Introduction

Aging is a multifactorial process, which is characterized by the gradual loss of physiological integrity of organs, leading to severe organ vulnerability, impairing body function until death (Blagosklonny, 2012; Manzano-Crespo et al., 2019). The intestine is the main organ for the body to absorb nutrients, and it is also an effective barrier to prevent harmful substances such as bacteria and viruses from entering the body (Wiles et al., 2020). In the aging process, intestine is one of the organs of the body that begins to age (Ogra, 2010). In the process of intestinal aging, there are intestinal tissue degeneration, increased inflammation, thickening of muscle layer, weakened intestinal contraction and peristalsis, and decreased levels of various digestive enzymes, leading to intestinal digestion and absorption function decline, and even chronic constipation and defecation disorders often occur (Lewis et al., 2020; Özsoy et al., 2020). Nowadays, the average human life in many countries of the world has increased unprecedentedly, the problem of international population aging has become increasingly serious, and human fertility has also declined rapidly (Weinberger, 2018). However, its mechanismic role maintains unclear. Therefore, exploring the antiaging methods has become a research hotspot in the medical field.

Resveratrol is a non-flavonoid polyphenol compound, which is naturally exists in grapes, *Polygonum cuspidatum* and other plants, has a variety of pharmacological activities such as antioxidation, antiaging, antiinflammation, anticancer, antidiabetes, heart protection and nerve protection (Shaito et al., 2020; Tian and Liu, 2020). In addition, resveratrol is considered a classic antiaging compound, such as Jeong et al. (Yoon et al., 2020) reported that the addition of resveratrol could increase pregnancy outcomes in women of advanced maternal age. Huang et al. (Huang et al., 2020) showed that supplementary resveratrol improved most of the altered metabolic dysregulation and related dysbiosis programmed by maternal and postnatal high-fat diet exposure. However, the pharmacological mechanisms of resveratrol for intestinal aging are not fully understood.

Network pharmacology is a discipline that explains the process of disease development based on the principle of systems biology and the holistic view of network balance to understand the interaction between drugs and the body, which has the characteristics of “multi-gene, multi-target” (Chen et al., 2019; Li et al., 2019; Liu T. et al., 2020). From the perspective of network pharmacology, it is a potential research direction to systematically study the antiaging target of resveratrol by using a variety of database resources. The current study first studied the antiaging targets of resveratrol through network pharmacology, then enriched the targets with bioinformatics, and preliminarily screened the ATF4/Chop/Bcl-2/Bax signaling pathway in apoptosis as the research object. In addition, the collection of wild-type (WT) and *ATF4* knockdown type (KD) naturally aging mice was interfered with resveratrol to further verify the effects of resveratrol on ATF4/Chop/Bcl-2/Bax signaling pathway related proteins and mRNAs, as well as the effects on aging related indicators.

## Materials and Methods

### Construction of Target Library of Resveratrol

The targets of resveratrol were obtained by TCMSP (http://lsp.nwu.edu.cn/tcmsp.php, searched on November 5, 2019), and target library of resveratrol was constructed using WPS Office software 2019. Then the names of target proteins were transformed into the corresponding gene symbols by UniProt database (https://www.uniprot.org, searched on November 6, 2019).

### Acquisition of Aging Related Therapeutic Targets

The aging related therapeutic targets were obtained using GeneCards database (https://www.genecards.org, searched on November 6, 2019) and NCBI database (https://www.ncbi.nlm.nih.gov/gene, searched on June 22, 2020). The “Aging” was used to act as searching keyword. Then the putative targets of resveratrol and the known therapeutic targets on aging amalgamated.

### Network Construction of Resveratrol-Target-Aging Network and Protein-Protein Interaction Network

The resveratrol-target-aging network was conducted using Cytoscape_v3.7.1. And the protein-protein interaction PPI network was integrated and conducted using String database (https://string-db.org, updated on November 7, 2019) according to the common targets.

### Bioinformatics Analysis Based on Target Genes

Bioinformatics analysis based on target genes-related aging in resveratrol was performed using Database for Annotation, Visualization, and Integrated Discovery (DAVID) (https://david.ncifcrf.gov/, performed on November 7, 2019). Meanwhile, GO function and KEGG pathway enrichment analysis were performed.

### Animals and Experimental Protocols

Twenty 12-month-old female (wild type, WT) mice purchased from the Experimental Animal Center of Guangzhou University of Traditional Chinese Medicine, and sixteen twelve-month-old female *ATF4* ^+/-^ heterozygous (knock down, KD) mice established in Institute of model animals of Nanjing University using Cas9/RNA system gene targeting technology, were raised in the Experimental Animal Center of Jinan University. All mice used were in a C57BL/6 background. The use of *ATF4* ^+/−^ heterozygous mice was based on the fact that *ATF4* ^−/−^ null mice have been found to be mostly neonatal lethal, even surviving mice are dwarf and immunocompromised (Masuoka and Townes, 2002; Wang et al., 2009; Cornejo et al., 2013). The animal experimental study was approved by the Experimental Animal Ethics Committee of Jinan University, which conformed to the principles of animal protection, animal welfare and ethics, and the relevant provisions of national experimental animal welfare ethics (ethics number: 20191127-04). After 10 days of natural feeding, the current experiment was carried out. The two types of mice were respectively divided into control group and resveratrol group, with a total of four groups of 8–10 mice in each group. Resveratrol groups were conducted intragastric administration of 44 mg/(kg.day) resveratrol for 30 days, and control groups were conducted intragastric administration of equivalent saline. At the 31st day, all the mice were anesthetized by intraperitoneal injection of 0.3% sodium pentobarbital (30 mg/kg) to avoid suffering.

### Measurements the Expression of Superoxide Dismutase, Glutathione Peroxidase, Catalase, Linear Alkylethoxylate, and Malondialdehyde by ELISA

After anesthetized, the eyeball blood of the mice was collected in 5-ml blood collection tube. The intestinal tissues were cut along the longitudinal axis of the jejunal segment and washed three times with normal saline. Then the intestinal tissues were cut and put into different 1.5-ml EP tubes marked numbers, and then kept them into -80 °C. The eyeball blood were centrifuged with a freezing centrifuge, and the supernatants were taken into the 1.5-ml EP tube marked numbers. Before the measure, the intestinal tissues were homogenized separately. Finally, the contents of superoxide dismutase (SOD), glutathione peroxidase (GSH-Px), catalase (CAT), linear alkylethoxylate (AE), and malondialdehyde (MDA) in serum and intestinal tissues were detected by ELISA. The operational steps of the experiment were in accordance with the instructions of the kits (MeiMian, China).

### Observation of Pathological Changes in Intestinal Tissues by Hematoxylin and Eosin and Periodic Acid‐Schiff Staining

A mouse was randomly selected from each group and colon tissues were collected to conventionally immersed in 4% paraformaldehyde overnight under ordinary temperature, routinely dehydrated, embedded, prepared and stained with hematoxylin and eosin (HE) and Periodic acid-Schiff (PAS). Then they were observed under an optical microscope (microscope model: NIKON Eclipse ci, imaging system: NIKON digital sight DS-FI2, Japan). Each slice in each group was selected three fields of vision to take photos, and as many tissues as possible filled the whole field of vision to ensure that the background light of each photo was consistent. Then, Image-Pro Plus 6.0 (Media Cybernetics, Inc., Rockville, MD, USA) was used to count the number of goblet cells, the length of villi and the thickness of lamina propria on five intact villi.

### Observation of Apoptosis in Intestinal Tissues by Terminal Deoxynucleotidyl Transferase dUTP Nick End Labeling Staining

Paraffin embedding and sectioning were the repeat steps of the previous experiment. Paraffin sections were then dewaxed to water, repaired, broken, colored and examined, and photographed under the microscope. Finally, the apoptotic cells were analyzed with Image J software (National Institutes of Health, USA) and counted as IOD/Area. Transferase dUTP Nick End Labeling (TUNEL) kit (Roche; 11684817910; USA) was applied and the experimental procedures were according to the instructions of the kit.

### Measurements of ATF4/Chop/Bcl2/Bax Signaling Pathway-Related Proteins Expression by Western Blotting

Three mice were randomly selected from each group and the proteins in their intestinal tissues were detected using Western blotting. ATF4 (11815S; CST; USA), Chop (2895S; CST; USA), Bax (2764S; CST; USA), and Bcl-2 (D038-3; MBL; Japan) antibodies were used in this study. According to the kit instructions, the total proteins were obtained by conventional tissue lysis and their concentrations were measured. PAGE separation was performed after equivalent sampling. PAGE separins used 10% separating gel and 5% stacking gel. Proteins separated by PAGE were transferred to PVDF membranes and primary and secondary antibodies were added. The proteins were exposed, developed and fixed on X-ray film with ECL chemiluminescence reagent. GAPDH was used as an internal control. Quantity One 4.0 software was used to analyze the imaging map and calculate the ratios of ATF4, Chop, Bcl-2 and Bax proteins in each group.

### Measurement of ATF4/Chop/Bcl-2/Bax Signaling Pathway-Related Genes Expression by Real-Time PCR

Total RNA isolation was collected by means of Trizol reagent following the manufacturer’s instructions. Samples consisting of 2-μl total RNA from each group were digested with RNase-free DNase I (Thermo Fisher Scientific) and then reverse transcribed into cDNA using Transcriptor reverse transcriptase (Takara, Japan). Real-time PCR analysis was performed in triplicate on a CFX96 real-time PCR system (Bio-Rad) at a final volume of 20 μl. Each reaction contained 10-μl 2x SYBR Green qPCR SuperMix (invitrogen), 5-μl cDNA, 4-μl ddH2O, and 1-μl primer mix. In addition, the Gapdh gene was used for the housekeeping control. The PCR primers are listed in **Supplementary Table 1**.

### Statistical Analysis

All the data were expressed as means ± standard error of the mean (SEM). A practical t-test method for comparison between two groups was conducted and statistical charts were created using GraphPad Prism software. If *p* values were less than 0.05, the difference was considered as statistically significant.

## Results

### Network Pharmacology Revealed the Targets Characteristics of Resveratrol for Aging

**Figure 1:**
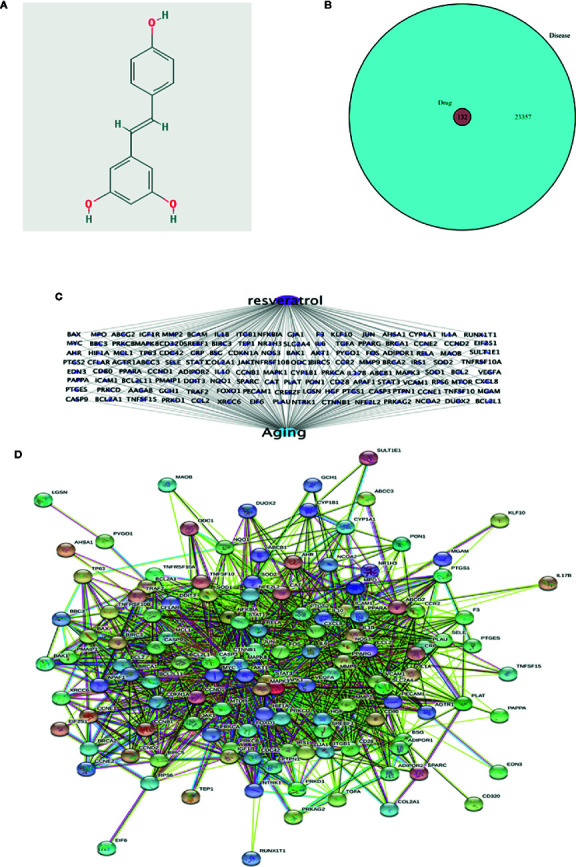
Network pharmacology revealed the targets characteristics of resveratrol for aging. **(A)** Molecule structure of resveratrol. **(B)** Venn diagram of common targets of resveratrol for aging. **(C)** resveratrol-target-aging network. **(D)** The protein-protein interaction (PPI) network based on targets of resveratrol on aging. Nodes represent different proteins. Edges represent protein-protein associations, the line thickness indicates the strength of data support.

Network pharmacology of resveratrol for aging was shown in **Figure 1**. Molecular formula of resveratrol was C14H12O3, and the molecule structure was shown as **Figure 1A**. A total of 132 targets of resveratrol were obtained by TCMSP and converted into symbols of corresponding genes using UniProt (**Supplementary Table 2**). A total of 23489 aging-related targets were obtained from the databases (**Supplementary Table 3**). Combining the targets of resveratrol with aging-related targets, all the 132 targets of resveratrol were commonly found to be aging-related targets (**Figure 1B**; **Supplementary Table 4**). The resveratrol-target-aging network was conducted and shown as **Figure 1C**. The PPI network was conducted and the top 10 core genes were screened as *AKT1*, *IL6*, *VEGFA*, *CASP3*, *JUN*, *MAPK3*, *MAPK8*, *MYC*, *STAT3* and *MAPK1* (**Figure 1D**; **Supplementary Table 5**).

### Bioinformatics Analysis Based on Target Genes

**Figure 2:**
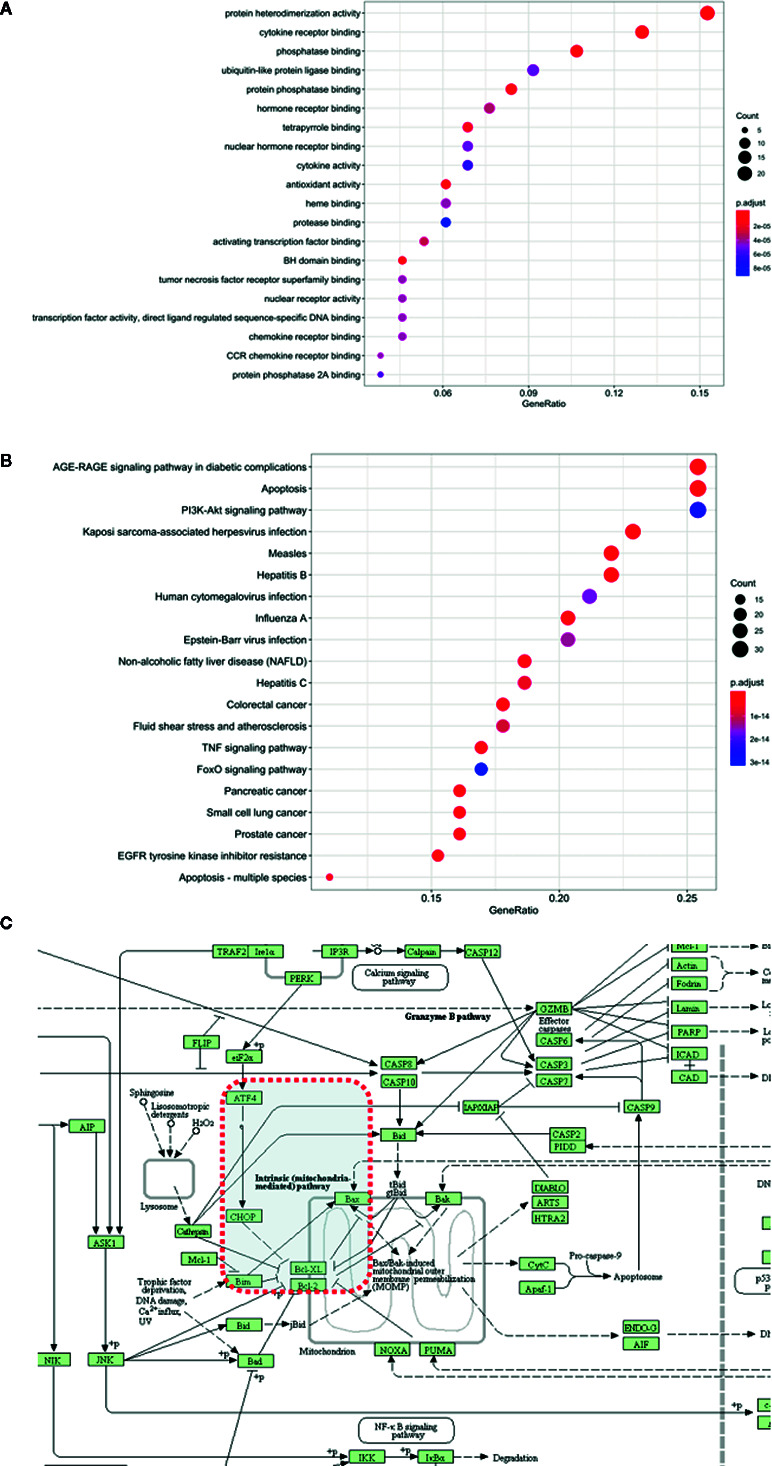
Bioinformatics analysis based on target genes. **(A)** GO enrichment pathway. **(B)** KEGG enrichment pathway. (The abscissa is the rich factor, the ordinate is pathway name, the number of genes is represented by the size of the dot and the color represents the *p* value). **(C)** Screening of ATF4/Chop/Bcl-2/Bax signaling pathway in apoptosis.

Enrichment analysis is a visualization process that specifies the biological functions of related genes and proteins. The biological functions involved in predicting core targets in biological processes are systematically elucidated. In this study, GO enrichment analysis was performed for 132 genes of resveratrol, and the top 20 enrichment data were plotted as a bubble chart (**Figure 2A**; **Supplementary Table 6**). Biological functions involved mainly include protein heterodimerization activity, cytokine receptor binding, phosphatase binding, and protein phosphatase binding process, etc. For these 132 targets, enrichment analysis of action pathway was performed based on the KEGG pathway database. It was found that the AGE-RAGE signaling pathway in diabetic complications, apoptosis, PI3K-Akt signaling pathway and other major signaling pathways are closely related to the targets of resveratrol for aging (**Figure 2B**; **Supplementary Table 7**). Based on the enriched pathway of apoptosis, the ATF4/Chop/Bcl-2/Bax signaling pathway was obtained through further screening in the apoptotic mechanism (**Figure 2C**).

### Effects of Resveratrol on Aging-Related Indicators in Serum

**Figure 3:**
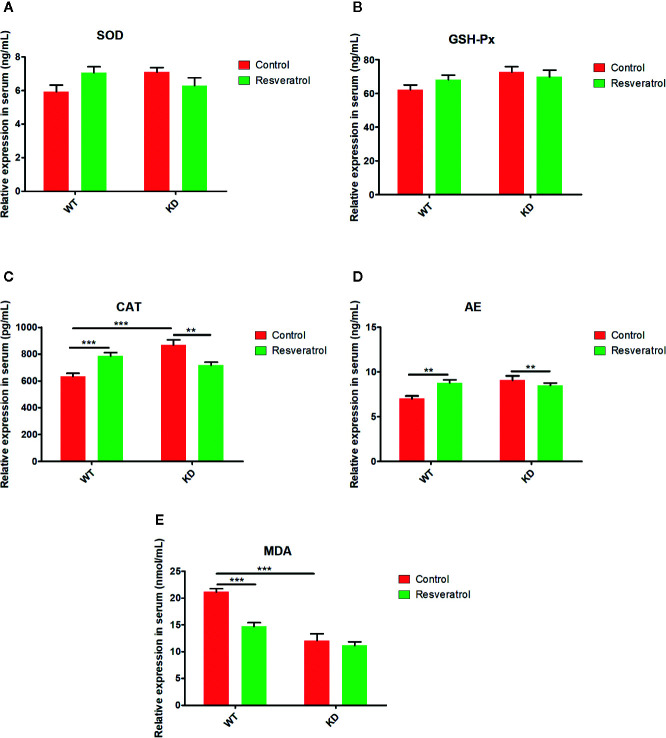
Effects of resveratrol on aging-related indicators in serum. **(A)** superoxide dismutase (SOD). **(B)** glutathione peroxidase (GSH-Px). **(C)** catalase (CAT). **(D)** linear alkylethoxylate (AE). **(E)** malondialdehyde (MDA). Mean ± Standard error of the mean (SEM) performed are represented. **p < 0.01, ***p < 0.001.

In order to further study the effects of resveratrol on aging, we detected the SOD, GSH-Px, CAT, AE, and MDA in serum which were considered as aging-related indicators (Zhang et al., 2019). In WT mice, it was found that resveratrol evidently upregulated the relative expression of SOD, GSH-Px, CAT, AE, whereas downregulated the relative expression of MDA in serum; however, there was no statistical difference in SOD and GSH-Px (**Figures 3A–E**). Moreover, it was obviously found that there was higher relative expression of SOD, GSH-Px, CAT, AE and lower relative expression of MDA in KD mice than that in WT mice; however, there was no statistical difference in SOD, GSH-Px, AE (**Figures 3A–E**). Additionally, in KD mice, it was found that resveratrol slightly downregulate the relative expression of CAT and AE; there was no evidently difference in SOD, GSH-Px, MDA in serum (**Figures 3A–E**).

### Effects of Resveratrol on Aging-Related Indicators in Intestinal Tissue

**Figure 4:**
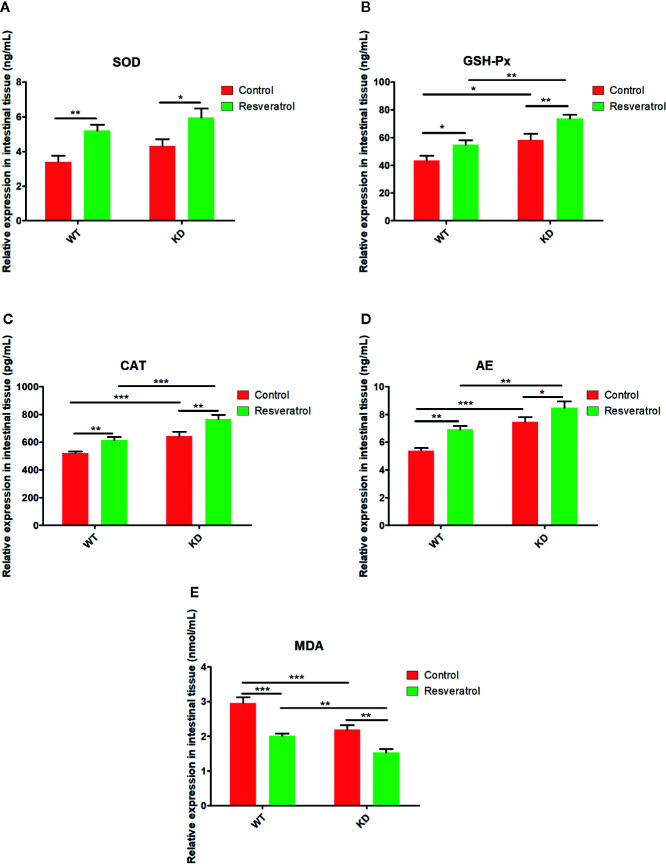
Effects of resveratrol on aging-related indicators in intestinal tissue. **(A)** superoxide dismutase (SOD). **(B)** glutathione peroxidase (GSH-Px). **(C)** catalase (CAT). **(D)** linear alkylethoxylate (AE). **(E)** malondialdehyde (MDA). Mean ± Standard error of the mean (SEM) performed are represented. *p < 0.05; **p < 0.01, ***p < 0.001.

Focusing on the aging of small inteatine, we tested the above indicators in the small intestine. In WT and KD mice, it was found that resveratrol statistically upregulated the relative expression of SOD, GSH-Px, CAT, AE, whereas downregulated the relative expression of MDA in intestine (**Figures 4A–E**). Moreover, it was obviously found that there was higher relative expression of SOD, GSH-Px, CAT, AE and lower relative expression of MDA in KD mice than that in WT mice; however, there was no statistical difference in SOD (**Figures 4A–E**). Additionally, it was found that these was higher relative expression of SOD, GSH-Px, CAT, AE and lower relative expression of MDA in KD mice than that in WT mice after resveratrol treatment; however, there was no statistical difference in SOD (**Figures 4A–E**).

### Observation of Pathological Changes in Intestinal Tissue by HE and PAS Staining

**Figure 5:**
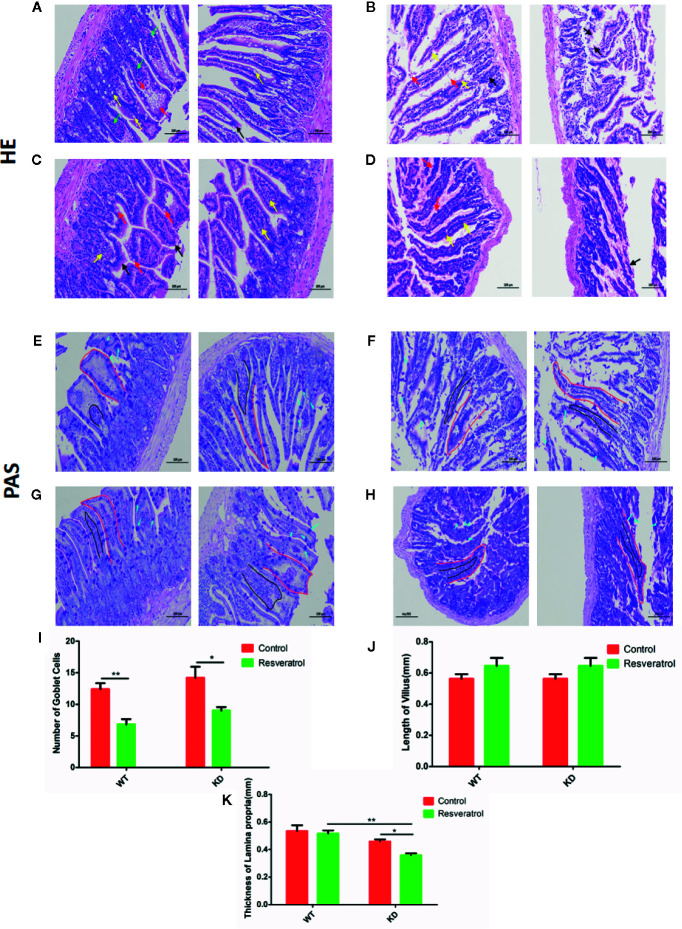
Observation of pathological changes in intestinal tissue by HE and PAS staining. HE staining: **(A–D)** **(A)** WT-control, **(B)** WT-resveratrol, **(C)** KD-control, **(D)** KD-resveratrol. villi (black arrow), purple flocculent (red arrow), goblet cells (yellow arrow), lamina propria (green arrow). PAS staining: (E–H), **(E)** WT-control, **(F)** WT-resveratrol, **(G)** KD-control, **(H)** KD-resveratrol. villi (red), goblet cells (blue), lamina propria (black). **(I)** number of goblet cell, **(J)** length of villi, **(K)** thickness of lamina propria. Mean ± Standard error of the mean (SEM) performed are represented. *p < 0.05; **p < 0.01.

HE and PAS staining were conducted to investigate the effects of resveratrol on aging-related intestinal tissue. Decreased number of goblet cell and thickness of lamina propria were determined in WT and KD mice after resveratrol treatment, but increased length of villi was found; there was no statistical difference in lamina propria and length of villi in WT mice (**Figures 5A–K**). Furthermore, lower thickness of lamina propria was found in KD mice than that in WT mice, but higher number of goblet cell was found, and there was no obviously different in length of villi; however, there was no statistical significant changes in thickness of lamina propria and number of goblet cell (**Figures 5A–K**). It was found that these was lower thickness of lamina propria and higher number of goblet cell in KD mice than that in WT mice after resveratrol treatment and no obviously different in length of villi; however, there was no statistical difference in number of goblet cell (**Figures 5A–K**).

### Effects of Resveratrol on Apoptosis in Intestinal Tissues by TUNEL Staining

**Figure 6:**
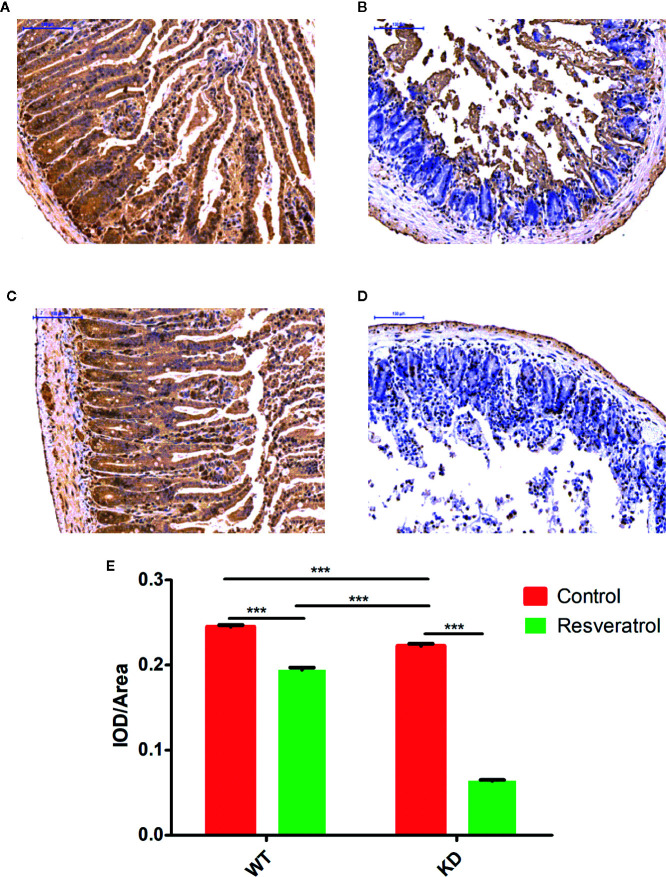
Effects of resveratrol on apoptosis in intestinal tissue by transferase dUTP nick end labeling (TUNEL) staining. **(A–D)** **(A)** WT-control, **(B)** WT-resveratrol, **(C)** KD-control, **(D)** KD-resveratrol. Brown indicates the number of apoptotic cells, and the darker the color is, the more apoptotic the cells are. **(E)** IOD/Area. Mean ± standard error of the mean (SEM) performed are represented. ***p < 0.001.

The TUNEL staining used to identify the apoptotic cells in intestinal tissues. Decreased apoptotic cells were determined in WT and KD mice after resveratrol treatment (**Figures 6A–E**). Furthermore, lower apoptotic cells were found in KD mice than that in WT mice (**Figures 6A–E**). Additionally, it was found that these were lower apoptotic cells in KD mice than that in WT mice after resveratrol treatment (**Figures 6A–E**).

### Effects of Resveratrol on ATF4/Chop/Bcl-2/Bax Signaling Pathway-Related Proteins Expression in Intestinal Tissue

**Figure 7:**
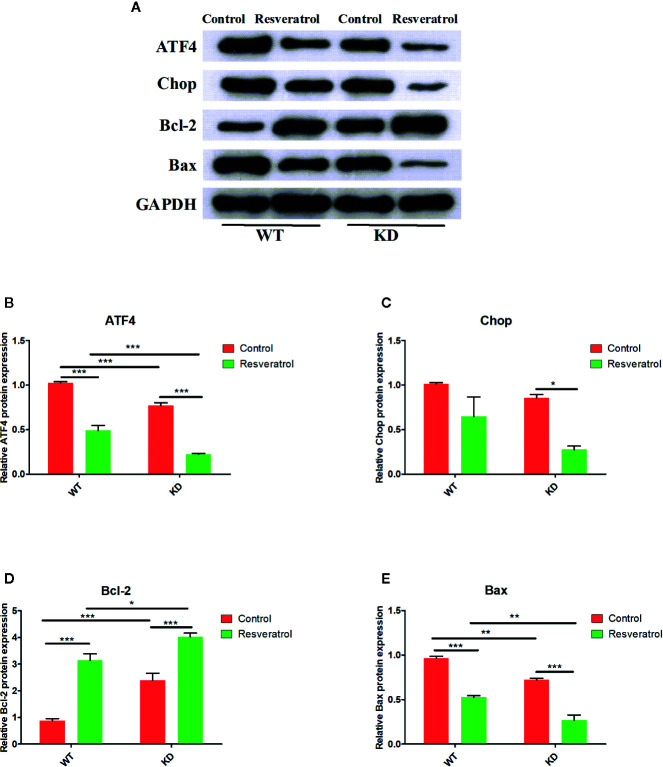
Effects of resveratrol on ATF4/Chop/Bcl-2/Bax signaling pathway-related protein expression in intestinal tissue. **(A)** The western blotting of indicated proteins of ATF4, Chop, Bcl-2 and Bax. **(B)** The quantification of ATF4 protein. **(C)** The quantification of Chop protein. **(D)** The quantification of Bcl-2 protein. **(E)** The quantification of Bax protein. Protein levels were messured by Western blot. Mean ± Standard error of the mean (SEM) performed in triplicate are represented. *p < 0.05; **p < 0.01, ***p < 0.001.

To further study the effects of resveratrol on ATF4/Chop/Bcl-2/Bax signaling pathway, we detected the key proteins of ATF4, Chop, Bcl-2, Bax in the signaling pathway. Decreased ATF4, Chop, Bax proteins expression were determined in WT and KD mice after resveratrol treatment, but increased Bcl-2 proteins expression were found; there was no statistical difference in Chop in WT mice (**Figures 7A–E**). Furthermore, lower ATF4, Chop, Bax proteins expression were found in KD mice than that in WT mice, but higher Bcl-2 proteins expression were found; there were no statistical significant changes in Chop (**Figures 7A–E**). It was found that these was lower relative protein expression of ATF4, Chop, Bax and higher relative expression of Bcl-2 in KD mice than that in WT mice after resveratrol treatment; however, there was no statistical difference in Chop (**Figures 7A–E**).

### Effects of Resveratrol on ATF4/Chop/Bcl-2/Bax Signaling Pathway-Related Genes Expression in Intestinal Tissue

**Figure 8:**
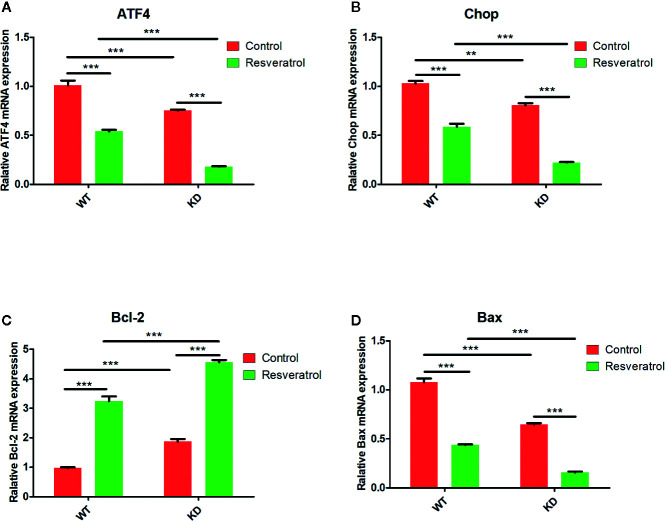
Effects of resveratrol on ATF4/Chop/Bcl-2/Bax signaling pathway-related gene expression in intestinal tissue. **(A)** Gene expression for ATF4. **(B)** Gene expression for Chop. **(C)** Gene expression for Bcl-2. **(D)** Gene expression for Bax. Gene expression levels were messured by real-time PCR. Mean ± Standard error of the mean (SEM) performed in triplicate are represented. **p < 0.01, ***p < 0.001.

In order to better study the effects of resveratrol on ATF4/Chop/Bcl-2/Bax signaling pathway, the key genes (ATF4, Chop, Bcl-2, Bax) related to the signaling pathway in mRNA levels were detected. Significantly decreased ATF4, Chop, Bax mRNAs expression were determined in WT and KD mice after resveratrol treated, but increased Bcl-2 mRNAs expression were found (**Figures 8A–D**). Moreover, lower ATF4, Chop, Bax mRNAs expression were significantly found in KD mice than that in WT mice, but higher Bcl-2 mRNAs expression were found (**Figures 8A–D**). It was found that these was lower relative mRNA expression of ATF4, Chop, Bax, and higher relative expression of Bcl-2 in KD mice than that in WT mice after resveratrol treated (**Figures 8A–D**). Obviously, the changes in protein and mRNA levels exhibited statistically significantly consistentence.

## Discussion

Aging, as a life phenomenon, is caused by genetic and environmental factors (Blagosklonny, 2012). Intestine is the body’s largest digestive organ, rich in many bacteria and enzymes etc., preventing the invasion of exogenous substances while absorbing nutrients (Ogra, 2010; Strilbytska et al., 2020; Wiles et al., 2020). Intestinal aging, often causes a series of chain reactions, such as intestinal inflammation, bacterial imbalance, immune deficiency, etc., which accelerate the intestinal aging (Kühn et al., 2020; Rampelli et al., 2020). Recent studies suggested that aging is mainly related to oxidative stress, cell apoptosis, autophagy and intestinal flora (Chen Y. et al., 2020; Wang F. et al., 2020; Xu et al., 2020). Resveratrol is one of compounds to slow aging. Studies have shown that resveratrol attenuates hydrogen peroxide-induced aging by upregulating autophagy in human umbilical vein endothelial cells (Du et al., 2019). Li et al. have shown resveratrol delay age-related cognitive decline through SIRT1 which the regulatory mechanisms include antioxidative, antiinflammatory, antiapoptotic processes, and autophagy regulation, as well as increases in cerebral blood flow and improvements in the plasticity of synaptic pathways (Cao et al., 2018). However, the mechanism of its pharmacological action has not been fully understood.

Previous studies have provided clues for this study, which first discussed the antiaging mechanism of resveratrol with the help of network pharmacology. Network pharmacology in understanding “aging-targets-resveratrol” interaction network on the basis of the analysis through the network to observe resveratrol intervention and influence of complex pathological Internet use visualization of large-scale data integration intuitive, clear observation network interactions between the various nodes, provide a new platform for the research on the mechanism of aging targets. The results of network pharmacology showed that 132 targets of resveratrol were all aging targets. According to the above data, the network map of resveratrol - target - disease visualizes the intersection target of resveratrol and disease. The core targets of the top 10 (AKT1, IL6, VEGFA, CASP3, JUN, MAPK3, MAPK8, MYC, STAT3, and MAPK1) were identified by String database analysis of gene interaction. The enrichment analysis results of GO and KEGG showed that resveratrol antiaging involved mainly included protein heterodimerization activity, cytokine receptor binding, phosatase binding, and protein phosatase binding process were found. The main pharmacological pathways include antiapoptosis, PI3K-Akt signaling pathway, etc. The ATF4/Chop/Bcl-2/Bax signaling pathway was obtained through further screening in the apoptotic mechanism. Network pharmacology reveals the complex antiaging mechanism of resveratrol through multi-target, multi-pathway and multi-pathway, and discusses the potential antiaging target of resveratrol, which provides clues and reference directions for the follow-up animal experiments, and lays a good foundation for further discussion of the antiaging pharmacological target and molecular mechanism of resveratrol.

Aging is closely related to oxidative stress, endoplasmic reticulum stress and apoptosis (Chen G. et al., 2020; Kandlur et al., 2020). SOD, GSH-Px, CAT, AE, MDA are considered to be related to oxidative stress, and they were found that SOD, GSH-Px, CAT, AE, MDA worked as important parameters reflecting the potential antiaging ability of the body by many previous reasearches (Akbulut et al., 2009; Hui et al., 2020; Wang L. et al., 2020; Song et al., 2020). The results showed that resveratrol did have the effect of delaying intestinal aging related indexes, which further revealed that resveratrol delayed intestinal aging. Also, these results in the blood could indicate this, although the differences were not statistically significant in the blood compared to the intestinal tissue. We thought that intestinal aging might be an early lesion site of aging, leading to the preceding differences. HE and PAS staining results could also visually show resveratrol’s role in protecting intestinal tissue from aging. However, the number of goblet cells decreased due to resveratrol administration was found in this study. Goblet cells can promote mucus secretion and increase the thickness of mucus barrier, thus enhancing resistance and defense ability (Cortez et al., 2020). In order to increase the thickness of mucus barrier and defense, resveratrol supplementation for a long time might cause mucus secretion of goblet cells, so to a certain extent, goblet cells were consumed. Therefore, the number of goblet cells decreased. Meanwhile, the results showed that the thickness of lamina propria was reduced in KD mice. There are lymphocytes in the lamina propria, which are the effective sites of immune protection function of intestinal mucosal immune system and have the function of immune defense (Ge et al., 2020). Compared with WT mice, there was less intestinal aging and needing less resveratrol in KD mice. Long-term supplement of resveratrol might increase the intestinal barrier pressure of KD mice, so as to prevent foreign resveratrol. Therefore, the lamina propria might be consumed and reduced in KD mice. Additionally, the results of TUNEL staining showed that there were inhibitory effects of resveratrol on apoptosis in intestinal tissues. Therefore, these findings illustrated resveratrol could inhibit apoptosis and delay intestinal aging in mice.

ATF4/Chop/Bcl-2/Bax signaling pathway was selected for research based on bioinformatics analysis. Evidence have suggested that ATF4/Chop/Bcl-2/Bax signaling pathway is involved in multiple metabolic functions of the body, such as endoplasmic reticulum stress, apoptosis, autophagy, ect (Yoneshima et al., 2016; Liu H. et al., 2020; Sharifi et al., 2020; Zeng et al., 2020). The expression of SOD, GSH-Px, CAT, AE in antioxidant enzymes and the level of ATF4, CHOP, BCL-2, and BAX in ATF4/Chop/Bcl-2/Bax signaling pathways were simultaneously found aberrant expression in endoplasmic reticulum stress and apoptosis (Gao et al., 2017; Xiao et al., 2019; Zhao et al., 2019; Li et al., 2020). Moreover, it was found that NaHS restored the decreased activity of antioxidant enzymes such as GSH, SOD, and CAT in the aging model by regulating to PI3K/AKT signaling pathways (Chen et al., 2017). Many modern studies have shown that the expression of the key genes (ATF4, Chop, Bcl-2, Bax) in ATF4/Chop/Bcl-2/Bax signaling pathway are closely related to the sirtuin activation (Yu et al., 2015; Yu et al., 2017). Such as the expression of ATF4 and Chop mRNA were exacerbated by Sirtuin-1 (SIRT1) inhibition, indicating that the sirtuin activation may related to ATF4/Chop/Bcl-2/Bax signaling pathway (Chan et al., 2017). Liu et al. found SIRT1 overexpression ameliorated aged MSCs senescent phenotype and increases in Bcl-2/Bax ratio in ATF4/Chop/Bcl-2/Bax signaling pathway at protein level (Liu et al., 2014). In the current study, decreased ATF4, Chop, Bax but increased Bcl-2 proteins and mRNAs expression were determined after resveratrol treated, which revealed resveratrol could inhibit ATF4/Chop/Bcl-2/Bax signaling pathway in aging mice. Moreover, lower ATF4, Chop, Bax but higher Bcl-2 proteins and mRNAs expression were found in KD mice than that in WT mice, which illustrated the inhibition of ATF4 could inhibit ATF4/Chop/Bcl-2/Bax signaling pathway, and ATF4/Chop/Bcl-2/Bax signaling pathway might be related to intestinal aging. Combined the difference of SOD, GSH-Px, CAT, AE, MDA in KD mice and WT mice, it was displayed that the inhibition of ATF4/Chop/Bcl-2/Bax signaling pathway might delay intestinal aging. And the difference of HE staining between KD mice and WT mice also demonstrated ATF4/Chop/Bcl-2/Bax signaling pathway might be related to intestinal aging. Additionally, lower relative proteins and mRNAs expression of ATF4, Chop, Bax and higher relative expression of Bcl-2 in KD mice than that in WT mice after resveratrol treatment, which exhibited ATF4/Chop/Bcl-2/Bax signaling pathway was more significant inhibited in KD mice after resveratrol treated. There was higher relative expression of SOD, GSH-Px, CAT, AE and lower relative expression of MDA in KD mice than that in WT mice after resveratrol treatment, which discovered the inhibition of ATF4/Chop/Bcl-2/Bax signaling pathway could promote resveratrol to delay intestinal aging. Therefore, all of these findings revealed that intestinal aging was related to ATF4/Chop/Bcl-2/Bax signaling pathway and resveratrol could delay intestinal aging in mice by inhibiting ATF4/Chop/Bcl-2/Bax signaling pathway.

**Figure 9:**
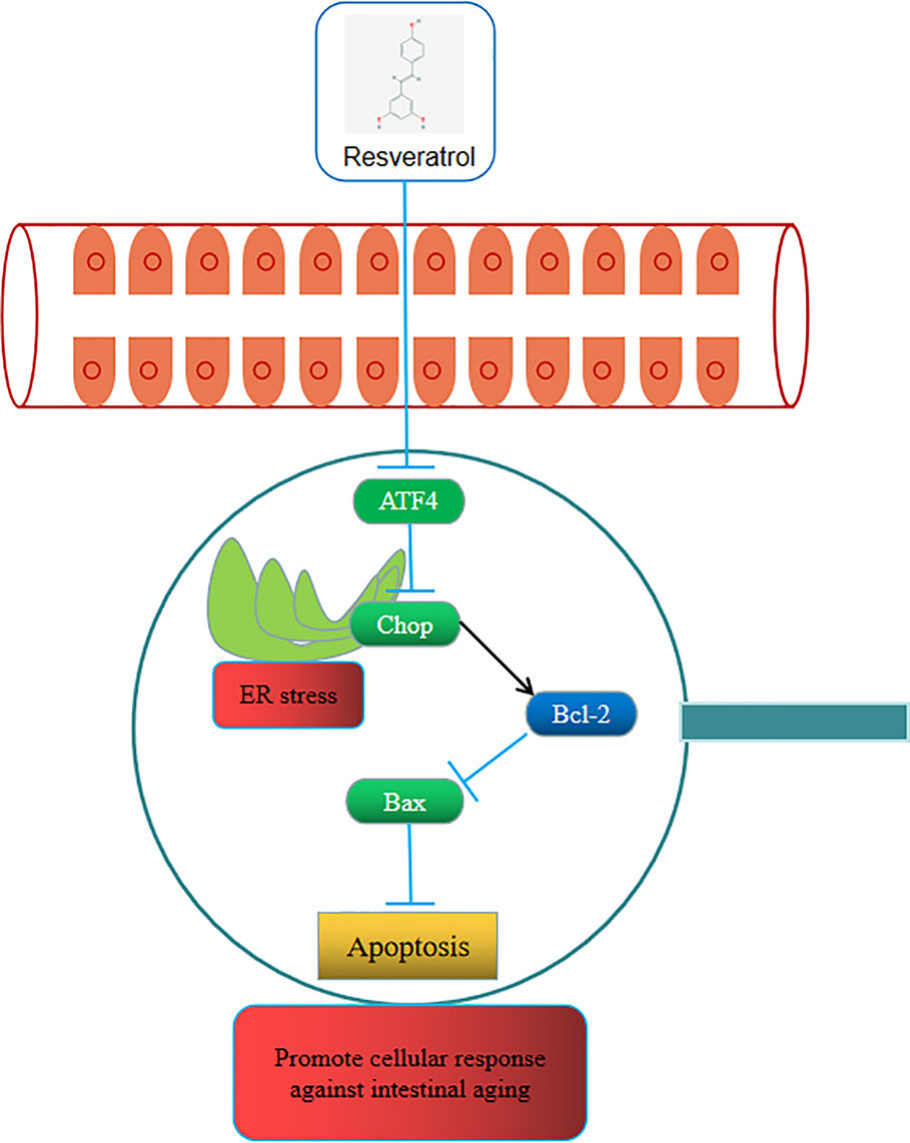
Representative cartoon with the potential mechanisms of resveratrol in aging mice.

Up to now, many studies confirmed that the mechanismic role of resveratrol on aging (Liu et al., 2018; Wiedenhoeft et al., 2019). Ran et al. focused on age-related changes in GI physiology and function, and thought the changes of the intestinal microbiota were related to aging and frailty (An et al., 2018). Moreover, phosphorylated ACT-5 accelerated decay of the intestinal subapical terminal web and impaired its interactions with cell junctions (Egge et al., 2019). Even Sung et al. showed the resveratrol-mediated changes in the gut microbiome might play an important role in the mechanism of action of resveratrol (Sung et al., 2017). However, the potential mechanisms of resveratrol on intestinal aging have not yet been fully investigated. To further understand the pharmacological mechanisms of resveratrol as a therapy for intestinal aging, in this study, we explore the potential mechanisms of resveratrol as a therapy against aging by compound-target network construction, PPI network analysis, GO enrichment analysis and KEGG enrichment analysis *via* network pharmacology approach. A previous study of network pharmacology showed anticolorectal cancer targets of resveratrol and biological molecular mechanism (Li et al., 2019). In our current study we found that the antiaging of resveratrol might be related to these biological processes containing regulation of apoptosis, ATF4/Chop/Bcl-2/Bax signaling pathway in biological processe of apoptosis was selected to verify the potential mechanisms. And ATF4 knockdown mice were used to conduct the inhibition of ATF4/Chop/Bcl-2/Bax signaling pathway to further verify its role in aging mice. Therefore, representative cartoon with the potential mechanism of resveratrol in aging mice was shown in **Figure 9**. Although such a design further explored the mechanismic role of resveratrol on intestinal aging, further mechanisms still need to be better studied.

## Conclusion

In summary, this study applied a network pharmacology approach documenting how resveratrol alter different pathways against aging, which is supplementary to other studies on resveratrol against aging. Moreover, a further animal experiment demonstrated that resveratrol substantially inhibited intestinal aging *via* downregulating ATF4/Chop/Bcl-2/Bax signaling pathway.

## Data Availability Statement

All datasets presented in this study are included in the article/**Supplementary Material**.

## Ethics Statement

The animal experimental study was approved by the Experimental Animal Ethics Committee of Jinan University, which conformed to the principles of animal protection, animal welfare and ethics, and the relevant provisions of national experimental animal welfare ethics (ethics number: 20191127-04).

## Author Contributions

LC, T-HL and YX participated in study design, W-CT, Q-EL, and W-QT searched databases, conduct animal experiment operation. LC, T-HL and YX helped to draft the manuscript. T-HL and YX carried out the statistical analysis of data.

## Funding

The study was supported by the National Natural Sciences Foundation of China (81673848 and 81603520), the Science and Technical Plan of Guangzhou, Guangdong, China (201707010100, 201804010213), the Natural Sciences Foundation of Guangdong Province (2017A030313658), the Administration of Traditional Medicine of Guangdong Province (20181068).

## Conflict of Interest

The authors declare that the research was conducted in the absence of any commercial or financial relationships that could be construed as a potential conflict of interest.

## Abbreviations

AE, linear alkylethoxylate; CAT, catalase; DAVID, Database for Annotation, Visualization and Integrated Discovery; GSH-Px, glutathione peroxidase; MDA, malondialdehyde; PPI, protein-protein interaction; SOD, superoxide dismutase.
